# Adherence to fast track measures in colorectal surgery—a survey among German and Austrian surgeons

**DOI:** 10.1007/s00384-023-04379-9

**Published:** 2023-03-25

**Authors:** Maria A. Willis, Peter S. Keller, Nils Sommer, Franziska Koch, Jörg-Peter Ritz, Katharina Beyer, Christoph Reißfelder, Julia Hardt, Alexander Herold, Heinz J. Buhr, Klaus Emmanuel, Joerg C. Kalff, Tim O. Vilz

**Affiliations:** 1 Department of Surgery, University Hospital Bonn, Campus 1, 53127 Bonn, Germany; 2 Department of General and Abdominal Surgery, Helios Hospital Schwerin, Schwerin, Germany; 3 Department of Surgery, Charité-Universitätsmedizin Berlin, Campus Benjamin Franklin, Berlin, Germany; 4 Department of Surgery, Medical Faculty Mannheim, Universitätsmedizin Mannheim, Heidelberg University, Heidelberg, Germany; 5 German Society of Coloproctology, Freiburg, Germany; 6 German Society of General- and Abdominal Surgeons, Germany, Berlin, Germany; 7 Department of Surgery, University Hospital Salzburg, Salzburg, Austria

**Keywords:** Fast track surgery, ERAS, Perioperative care, Colorectal surgery, Evidence-based medicine, Compliance

## Abstract

**Purpose:**

The effectiveness of modern perioperative treatment concepts has been demonstrated in several studies and meta-analyses. Despite good evidence, limited implementation of the fast track (FT) concept is still a widespread concern. To assess the status quo in Austrian and German hospitals, a survey on the implementation of FT measures was conducted among members of the German Society of General and Visceralsurgery (DGAV), the German Society of Coloproctology (DGK) and the Austrian Society of Surgery (OEGCH) to analyze where there is potential for improvement.

**Methods:**

Twenty questions on perioperative care of colorectal surgery patients were sent to the members of the DGAV, DGK and OEGCH using the online survey tool SurveyMonkey^®^. Descriptive data analysis was performed using Microsoft Excel.

**Results:**

While some of the FT measures have already been routinely adopted in clinical practice (e.g. minimally invasive surgical approach, early mobilization and diet buildup), for other components there are discrepancies between current recommendations and present implementation (e.g. the use of local nerve blocks to provide opioid-sparing analgesia or the use of abdominal drains).

**Conclusion:**

The implementation of the FT concept in Austria and Germany is still in need of improvement. Particularly regarding the use of abdominal drains and postoperative analgesia, there is a tendency to stick to traditional structures. To overcome the issues with FT implementation, the development of an evidence-based S3 guideline for perioperative care, followed by the founding of a surgical working group to conduct a structured education and certification process, may lead to significant improvements in perioperative patient care.

**Supplementary Information:**

The online version contains supplementary material available at 10.1007/s00384-023-04379-9.

## Background

Multimodal perioperative management (mPOM) concepts (also called enhanced recovery after surgery (ERAS) or fast track (FT) surgery) aim to accelerate recovery after surgery and to mitigate or prevent undesirable consequences such as infectious complications or postoperative intestinal motility disorders by various perioperative measures. The effectiveness of the FT concept, which has been continuously developed since its initial description by Kehlet in 1995, has already been demonstrated in various studies and meta-analyses [1–3]. However, it has been shown that a protocol adherence of more than 70% is required to achieve the desired effect [4]. If achieved, it might also lead to improved oncologic outcomes after resection of colorectal cancer, in addition to a reduction in complication rates and length of hospital stay (LOS) [2, 5].

Despite the good evidence for the effectiveness of FT measures, implementation is very hesitant. A multicenter observational study in 12 European hospitals in 2017 demonstrated an average protocol adherence of only 44% [6]. Among the most frequently stated reasons for failure are lack of human and financial resources, adherence to established, traditional treatment concepts and inadequate collaboration within the interdisciplinary team (anesthesia, surgery, nutritional medicine, nursing, physical therapy) [7]. Nevertheless, not only the implementation of the overall concept plays an important role, but also individual measures can decisively improve the short- and long-term outcome. This has been shown, for example, for prehabilitation, early enteral nutrition and opioid-sparing analgesia [8–10].

To determine the current status of fast-track adherence in German-speaking countries, we conducted a survey among members of the German Society for General and Visceral Surgery (DGAV), the German Society for Coloproctology (DGK) and the Austrian Society for Surgery (OEGCH).

## Methods

To assess the current status quo of perioperative management in colorectal surgery, a questionnaire consisting of 21 questions was developed (Supplement 1). Besides a few questions about the participants themselves (age, professional position and employer), this questionnaire mainly contained multiple- and single-choice questions about different perioperative measures. The questionnaire was created digitally with the online survey tool SurveyMonkey^®^ (www.surveymonkey.de). A link to the survey was then sent to the members of the DGAV, DGK and OEGCH via their mailing lists. In the period from January 25 to March 31, 2021, the questionnaire could be answered online. The statistical analysis of the data was performed with Microsoft Excel.

For the differentiated interpretation of the results depending on the professional position or the type of hospital, only answers from participants who are currently working at a hospital were taken into consideration. If a participant did not provide any information on these aspects, these questionnaires were not included in the differentiated evaluation, which led to possible deviations in the results compared to the overall collective.

## Results

A total of 233 surgeons participated in the survey. Information regarding age, professional position and field of activity is given in Table 1.

**Table 1:** Survey participants

**Survey participants**		**Percent**
**Age** **(*n* = 229)**	< 30 years	6.6% (*n* = 15)
	31–40 years	15.2% (*n* = 36)
	41–50 years	29.7% (*n* = 68)
	51–60 years	36.7% (*n* = 84)
	61–70 years	11.4% (*n* = 26)
**Professional position (*n* = 230)**	Resident	10.9% (*n* = 25)
	Attending physicians	6.5% (*n* = 15)
	Senior physician	25.7% (*n* = 59)
	Leading senior physician	11.3% (*n* = 26)
	Chief	44.8% (*n* = 103)
	In private practice	0.4% (*n* = 1)
	Others	0.4% (*n* = 1)
**Employer (*n* = 229)**	University Hospital	17.5% (*n* = 40)
	Tertiary care hospital	15.7% (*n* = 36)
	Secondary care hospital	28.4% (*n* = 65)
	Primary care hospital	37.6% (*n* = 86)
	Private practice	0.4% (*n* = 1)
	Other	0.4% (*n* = 1)

### Implementation of a perioperative FT treatment concept

Standard Operating Procedures (SOPs) are used to ensure uniform patient care and to enforce internal hospital standards; 89.3% of the respondents confirmed that a corresponding SOP or comparable exists at their hospital for perioperative patient care for colorectal surgery. However, implementation of a standardized FT treatment concept was reported by only 67.4% of participants. Furthermore, the existence of an interdisciplinary FT team consisting of at least surgical and anesthesia colleagues was reported by only 121 survey participants (51.9%). Additional support of this team by a nurse specialized in FT was reported by only 27 survey participants (11.6%).

A comparison of individual fast track elements and their implementation according to our survey is presented in Table 2.

**Table 2:** Overview of individual fast track elements and their implementation

**Fast track recommendation**	**Adherence**
Patient education about the fast track concept	88.0%
Prehabilitation *- Recommendation on nicotine abstinence* *- Recommendation on alcohol abstinence* *- Recommendation to increase physical activity* *- Optimization of nutritional intake/prevention of malnutrition*	18.9% 13.7% 36.9% 72.5%
Preoperative bowel preparation *- Before all elective colorectal resections* *- Only before rectal resections* *- No bowel preparation*	71.2% 16.7% 5.6%
Preoperative fasting	
*- Food intake up to 6 h before the start of surgery* *- Drinking clear liquids up to 2 h before surgery*	35.6% 48.5%
Predominantly minimally invasive surgical procedure (MIS)	
*- For colon resections*	80.3%
*- For rectal resections*	82.0%
Opioid-sparing analgesia	open/MIS*
*- Use of peridural catheters*	88.0%/58.8%
*- Use of a TAP block*	4.7%/6.4%
*- Local infiltration of the incision(s)*	15.0%/35.6%
Early removal of tubes/catheters *- Removal of gastric tubes postoperatively in the operating room* *- Removal of the urinary catheter before the 2*^*nd*^* postoperative day* *- Abandonment of routine abdominal drains*	Colon/rectal resection 82.0%/80.3% 56.7%/23.2% 33.5%/9.4%
Initial mobilization on the day of surgery	58.8%
Postoperative diet *- Liquid food postoperatively on the day of surgery* *- Solid diet from the first postoperative day onwards*	54.9% 32.6%

^*MIS: Minimally invasive surgery^

### Patient information and prehabilitation

The majority of respondents state that their hospital provides special education about the perioperative treatment concept prior to colorectal surgery. In 58.8% of hospitals, this information is part of the preoperative surgical risk education. However, 9.4% report that education is explicitly provided by a nurse specializing in FT, and 19.7% report the use of a patient brochure or informational videos.

Counseling or training on abstinence from nicotine or alcohol in preparation for planned surgery is provided in 18.9% (*n* = 44) and 13.7% (*n* = 32) of hospitals, respectively. Recommendations to increase physical activity are routinely provided by 36.9% (*n* = 86), according to respondents. In contrast, measures to prevent malnutrition or optimize nutritional intake are offered by 72.5% (*n* = 169).

Looking at the survey results differentiated by professional position of the participants and type of hospital (Table 3), it becomes apparent that the least support for preoperative measures is offered in primary and secondary care hospitals. It is also striking that it is mainly chief physicians who indicate the existence of such support in their hospital.

**Table 3:** Implementation of prehabilitation measures depending on the professional position and type of hospital

	**Recommendation on nicotine abstinence**	**Recommendation on alcohol abstinence**	**Recommendation to increase physical activity**	**Optimization of nutritional intake/prevention of malnutrition**
**Resident (*n*** **= 25)**	12.0% (*n* = 3)	16.0% (*n* = 4)	32.0% (*n* = 8)	64.0% (*n* = 16)
**Attending physicians (*n*** **= 15)**	13.3% (*n* = 2)	0.0% (*n* = 0)	13.3% (*n* = 2)	60.0% (*n* = 9)
**Senior physician (*n*** **= 59)**	11.9% (*n* = 7)	10.2% (*n* = 6)	22.0% (*n* = 13)	76.3% (*n* = 45)
**Leading senior physician (*n*** **= 26)**	7.7% (*n* = 2)	3.9% (*n* = 1)	30.8% (*n* = 8)	69.2% (*n* = 18)
**Chief (*n*** **= 103)**	29.1% (*n* = 30)	20.4% (*n* = 21)	52.4% (*n* = 54)	77.7% (*n* = 80)
**University Hospital (*n*** **= 40)**	20.0% (*n* = 8)	12.5% (*n* = 5)	27.5% (*n* = 11)	80.0% (*n* = 32)
**Tertiary care hospital (*n*** **= 36)**	22.2% (*n* = 8)	19.4% (*n* = 7)	41.7% (*n* = 15)	83.3% (*n* = 30)
**Secondary care hospital (*n*** **= 65)**	21.5% (*n* = 14)	16.9% (*n* = 11)	55.4% (*n* = 36)	83.1% (*n* = 54)
**Primary care hospital (*n*** **= 86)**	16.3% (*n* = 14)	10.5% (*n* = 9)	26.7% (*n* = 23)	59.3% (*n* = 51)

### Perioperative bowel preparation

Of the respondents, 5.6% (*n* = 13) reported that no routine bowel preparation is performed at their hospital prior to colorectal resections. While 16.7% report that bowel preparation is only performed prior to rectal procedures, the majority (71.2%, *n* = 166) report that bowel preparation is performed prior to every elective colorectal resection. In 53.6% of cases, this is performed as a combined mechanical and oral antibiotic bowel preparation. However, mechanical bowel lavage alone (33.9%, *n* = 79) or sole oral antibiotic bowel preparation (2.1%, *n* = 5) is also used (Table 4).

**Table 4:** Type of preoperative bowel preparation

**Type of preoperative bowel preparation**	**Percent**
Sole mechanical bowel preparation	33.9%
Sole oral antibiotic bowel preparation	2.1%
Combined mechanical and oral antibiotic bowel preparation	53.6%

Mechanical bowel preparation alone is relatively common in both primary care and university hospitals. However, combined mechanical and oral antibiotic bowel preparation is the predominant procedure at both university hospitals and secondary and tertiary care hospitals (Table 5).

**Table 5:** Preoperative bowel preparation depending on the type of hospital

	Sole mechanical bowel preparation	Sole oral antibiotic bowel preparation	Combined mechanical and oral antibiotic bowel preparation
**University Hospital (*n*** **= 40)**	45.0% (*n* = 18)	2.5% (*n* = 1)	50.0% (*n* = 20)
**Tertiary care hospital (*n*** **= 36)**	13.9% (*n* = 5)	0.0% (*n* = 0)	75.0% (*n* = 27)
**Secondary care hospital (*n*** **= 65)**	21.5% (*n* = 14)	3.1% (*n* = 2)	66.2% (*n* = 43)
**Primary care hospital (*n*** **= 86)**	45.4% (*n* = 39)	2.3% (*n* = 2)	40.7% (*n* = 35)

### Minimally invasive surgery (MIS)

For both colon and rectal resection, more than 80% of respondents indicated that minimally invasive techniques are predominantly used (that means a minimally invasive approach in > 50%) for the procedures in their hospital (Table 6).

**Table 6:** Predominantly used surgical approach

**Predominantly used surgical approach**	Main approach for colon resections (percent)	Main approach for rectal resections (percent)
Robotic resection	3.9%	20.6%
Laparoscopic surgery	76.4%	61.4%
Open surgery	15.5%	14.6%

*Looking at the answers of the respondents by hospital type and professional position in* *Table* *7* *, it can be seen that chief physicians and senior physicians indicate open surgery as the standard method to a lesser percentage than physicians in subordinate positions. Furthermore, it appears that especially at university hospitals, but also at tertiary care hospitals, robot-assisted surgery is frequently stated as the standard procedure for rectal resections. In contrast, robot-assisted surgery is only rarely indicated as a standard procedure in primary and secondary care hospitals.*

**Table 7:** Data on the predominantly used surgical procedure depending on the professional position and type of hospital

	Robotic resection		Laparoscopic surgery		Open surgery	
	Colon	Rectum	Colon	Rectum	Colon	Rectum
**Resident (** ***n*** ** = 25)**	0.0% (*n* = 0)	28.0% (*n* = 7)	64.0% (*n* = 16)	36.0% (*n* = 9)	36.0% (*n* = 9)	0.0% (*n* = 0)
**Attending physicians (** ***n*** ** = 15)**	6.7% (*n* = 1)	20.0% (*n* = 3)	53.3% (*n* = 8)	33.3% (*n* = 5)	40.0% (*n* = 6)	0.0% (*n* = 0)
**Senior physician (** ***n*** ** = 59)**	5.1% (*n* = 3)	25.4% (*n* = 15)	67.8% (*n* = 40)	54.2% (*n* = 32)	23.7% (*n* = 14)	5.1% (*n* = 3)
**Leading senior physician (** ***n*** ** = 26)**	7.7% (*n* = 2)	34.6% (*n* = 9)	88.5% (*n* = 23)	57.7% (*n* = 15)	3.9% (*n* = 1)	0.0% (*n* = 0)
**Chief (** ***n*** ** = 103)**	2.9% (*n* = 3)	13.6% (*n* = 14)	87.4% (*n* = 90)	79.6% (*n* = 82)	4.9% (*n* = 5)	1.9% (*n* = 2)
**University Hospital (** ***n*** ** = 40)**	5.0% (*n* = 2)	47.5% (*n* = 19)	60.0% (*n* = 24)	15.0% (*n* = 6)	35.0% (*n* = 14)	2.5% (*n* = 1)
**Tertiary care hospital (** ***n*** ** = 36)**	5.6% (*n* = 2)	38.9% (*n* = 19)	75.0% (*n* = 27)	58.3% (*n* = 21)	11.1% (*n* = 4)	2.8% (*n* = 1)
**Secondary care hospital (** ***n*** ** = 65)**	6.2% (*n* = 4)	16.9% (*n* = 11)	83.1% (*n* = 54)	73.9% (*n* = 48)	9.2% (*n* = 6)	1.5% (*n* = 1)
**Primary care hospital (** ***n*** ** = 86)**	1.2% (*n* = 1)	4.7% (*n* = 4)	82.6% (*n* = 71)	77.9% (*n* = 67)	12.8% (*n* = 11)	2.3% (*n* = 2)

### Postoperative analgesia

Postoperative analgesic therapy with intravenous or oral opioids is widely used for both open (61.8%; *n* = 144) and minimally invasive surgery (52.8%; *n* = 123). Another method used primarily after open surgery is the administration of local anesthetics via a peridural catheter (PDK) (88.0%; *n* = 205). However, this method is also regularly used after MIS, as reported by 58.8% of respondents (*n* = 137). Local infiltration of the surgical access or local nerve block (e.g. using transversus abdominis plane (TAP) block) are rarely used, regardless of the access route (Table 8) or the type of hospital (Table 9).

**Table 8:** Postoperative pain management measures used in open respectively minimally invasive procedures

**Mainly used measures for postoperative pain management**	**Percent** **open surgery**	**Percent** **MIS**
Epidural catheter	88.0%	58.8%
Transversus abdominis plane block	4.7%	6.4%
Local infiltration of the surgical access	15.0%	35.6%
Analgesic pump	42.9%	30.5%
Short infusions/oral administration of opioids	61.8%	52.8%
Analgesia alone using non-opioid analgesics	24.5%	32.6%

**Table 9:** Use of local analgesia procedures depending on the professional position and type of hospital

	**Local infiltration of the surgical access**		Transversus abdominis plane block	
	Open surgery	MIS	Open surgery	MIS
**Resident (*n* = 25)**	8.0% (*n* = 2)	36.0% (*n* = 9)	4.0% (*n* = 1)	12.0% (*n* = 3)
**Attending physicians (*n* = 15)**	13.3% (*n* = 2)	46.7% (*n* = 7)	0.0% (*n* = 0)	6.7% (*n* = 1)
**Senior physician (*n* = 59)**	8.5% (*n* = 5)	35.6% (*n* = 21)	1.7% (*n* = 1)	6.8% (*n* = 4)
**Leading senior physician (*n* = 26)**	15.4% (*n* = 4)	46.2% (*n* = 12)	7.7% (*n* = 2)	7.7% (*n* = 2)
**Chief (*n* = 103)**	20.4% (*n* = 21)	32.0% (*n* = 33)	6.8% (*n* = 7)	4.9% (*n* = 5)
**University Hospital (*n* = 40)**	15.0% (*n* = 6)	55.0% (*n* = 22)	0.0% (*n* = 0)	15.0% (*n* = 6)
**Tertiary care hospital (*n* = 36)**	13.9% (*n* = 5)	38.9% (*n* = 14)	2.8% (*n* = 1)	2.8% (*n* = 1)
**Secondary care hospital (*n* = 65)**	15.4% (*n* = 10)	30.8% (*n* = 20)	12.3% (*n* = 8)	9.2% (*n* = 6)
**Primary care hospital (*n* = 86)**	15.1% (*n* = 13)	29.1% (*n* = 25)	2.3% (*n* = 2)	2.3% (*n* = 2)

### Management of invasiveness

More than 80% of respondents reported that an intraoperatively inserted gastric tube is removed after completion of the surgical procedure before/at the end of anesthesia. Only 2.1% (*n* = 5) routinely leave the gastric tube in place through postoperative day (POD) 2.

The intraoperative urinary catheter is also removed within the first two postoperative days after colon resections (56.7%, *n* = 132). For rectal resections, the majority of respondents indicated that the urinary catheter is usually removed between POD 2 and 5 (65.2%, *n* = 152).

Regarding intraoperative placement of abdominal drains, 90.6% of respondents (*n* = 211) indicated that they routinely place drains after rectal resections. For colon resections, as many as 66.5% (*n* = 155) indicated that abdominal drains are placed routinely. There were no relevant variations in the answers of the respondents with regard to the type of hospital (Table 10). At just under 45%, secondary care hospitals are the most likely to forego routine drain placement for colon resections. Regarding professional position, chief physicians are more likely than subordinate physicians to refrain from routine drain placement for both colon and rectal resections.

**Table 10:** Omission of the routine insertion of abdominal drains depending on the professional position and type of hospital

	A**bandonment of the routine placement of abdominal drains**	
	Colon	Rectum
**Resident (*n* = 25)**	20.0% (*n* = 5)	8.0% (*n* = 2)
**Attending physicians (*n* = 15)**	33.3% (*n* = 5)	0.0% (*n* = 0)
**Senior physician (*n* = 59)**	25.4% (*n* = 15)	3.4% (*n* = 2)
**Leading senior physician (*n* = 26)**	34.6% (*n* = 9)	7.7% (*n* = 2)
**Chief (*n* = 103)**	42.7% (*n* = 44)	15.5% (*n* = 16)
**University Hospital (*n* = 40)**	27.5% (*n* = 11)	5.0% (*n* = 2)
**Tertiary care hospital (*n* = 36)**	33.3% (*n* = 12)	13.9% (*n* = 5)
**Secondary care hospital (*n* = 65)**	44.6% (*n* = 29)	13.9% (*n* = 9)
**Primary care hospital (*n* = 86)**	29.1% (*n* = 25)	7.0% (*n* = 6)

### Mobilization and diet

Of the respondents, 58.8% report that in their hospital, postoperative mobilization is started on the day of surgery; 39.1% begin mobilization on POD 1, and only one participant (0.4%) reports that postoperative mobilization does not begin until POD 2.

Regarding dietary buildup, 54.9% of the respondents state that their patients are allowed to eat liquid food on the evening of the surgery; 3.4% (*n* = 8) of the participants report that their patients receive solid food on the day of surgery. However, the majority of participants reported that their patients were allowed to resume solid food from the 1st POD (29.2%, *n* = 68) or 2nd POD (39.5%, *n* = 92). However, 26.6% (*n* = 62) of the survey participants indicated that the diet buildup does not begin until after the 2nd POD in their clinic. There were no differences between the respondents’ answers regarding the type of hospital or professional position.

## Discussion

In 2012, the ERAS Society published its first guideline on the optimal perioperative care of colorectal surgery patients including 20 recommendations [11]. In the past years, this concept has been continuously reviewed and developed, so that in the current version of the ERAS guideline, 25 items of perioperative care for colorectal surgery patients are assessed, and recommendations are made [12]. In the meantime, several national guidelines have also dealt with this topic [13–15]. However, despite the proven benefits of mPOM concepts, implementation is still hesitant.

To investigate the current status quo of adherence to mPOM concepts in German-speaking countries, we conducted a survey among the members of the DGAV, DGK and OEGCH. Although the results of this survey show that a large proportion of respondents state that a mPOM concept is implemented at their hospital, a closer look reveals that there is a considerable discrepancy between the existing recommendations on perioperative measures and their translation into clinical practice. Given the ever-present prominence of the topic in publications, lectures and at congresses, as well as the strong evidence for the effectiveness of the concept [2–4], mPOM enjoys a good acceptance and approval rate today. Unfortunately, this is not yet reflected in the implementation rate of the concept. The reasons for this as well as possible solutions are also the subject of ongoing discussions. SOPs, mPOM-specialized assistants and interdisciplinary teams that monitor the implementation of the concepts are, for example, among the measures intended to improve compliance [7, 16–18]. However, the results of our survey show that even if such structures already exist, this does not necessarily lead to a satisfactory implementation in the hospitals.

To enable good preparation for surgery, most of the respondents report that they provide specific information on perioperative management. And yet only a fraction of hospitals employs an assistant specialized in mPOM to take on this task. In times of limited time and human resources, online tutorials, videos or patient brochures are a possible alternative or, in the best case, complement to a mPOM assistant for patient education [19–21]. However, according to the results of our survey, even such means are only used relatively rarely.

Our survey also revealed potential for improvement regarding the performance of preoperative bowel preparation. Although there is still no uniform consensus on the most appropriate method, there is general agreement that purely mechanical bowel preparation is of no benefit [22]. The ERAS^©^ Society therefore recommends dispensing with bowel preparation altogether [12]. However, a recent Cochrane review on combined mechanical and oral antibiotic bowel preparation showed that combined bowel preparation, in contrast to purely mechanical bowel preparation, indeed leads to a reduction in infectious complications, whereas no conclusive assessment of the efficacy of oral antibiotic bowel preparation alone could be made due to insufficient data [23]. In contrast, our survey revealed that despite the lack of benefit, many hospitals continue to use sole mechanical bowel preparation, while sole oral antibiotic bowel preparation is hardly ever used.

Regarding the surgical approach, minimally invasive surgery (MIS) is considered the standard in colorectal surgery, which results in less surgical trauma and thus benefits patient recovery [24, 25]. Accordingly, more than 80% of the respondents stated that colon or rectal resections are predominantly performed minimally invasively in their hospital. But the predominance of the method is not enough. Data from the Federal Statistical Office show that the proportion of colon and rectal resections performed minimally invasively is only slightly higher than the proportion of open procedures. For instance, in 2020, 42% of low anterior rectal resections in Germany were performed open, and only 58% were initiated minimally invasively, with a conversion rate of 7% [26]. Considering the known advantages of minimally invasive procedures, the proportion of MIS should be much higher. So even though MIS is already predominantly used, there is still considerable potential for improvement.

Another contradiction between the supposed widespread implementation of the mPOM concept and everyday clinical practice can be seen in the still routine placement of abdominal drains. Despite good evidence [27], it has not yet been possible to eliminate the use of drains in colorectal surgery. The same applies to the use of modern analgesic concepts. According to our survey, besides oral opioids, peridural catheters are mainly used for analgesia. Newer methods, such as local nerve blocks like the TAP block or the intravenous application of lidocaine, are rarely used in Germany and Austria [28].

Another key in patient recovery after colorectal surgery is the transition to normal diet immediately after the operation. However, according to the respondents’ answers, there is still a delay in the postoperative buildup of the diet. This decision is not evidence-based, but “eminence-based”.

These are all examples that suggest that German and Austrian surgeons overestimate the implementation of mPOM concepts. Our survey confirms that there is a gap between the high acceptance level and the actual implementation of the concept.

A separate analysis of the data by hospital type shows that there is still a need for further improvement regardless of the treatment spectrum. It is known that a higher case volume leads to a better postoperative outcome [29, 30]. Therefore, one could assume that hospitals with a higher case volume have a more accomplished perioperative management. However, our study cannot confirm this assumption. While we did not measure case volume, if one assumes that the larger the hospital’s specialty, the larger the case volume, this would suggest that university hospitals and tertiary care hospitals would have to have a much more sophisticated mPOM concept than primary or secondary care hospitals. However, this is not the case.

The separate analysis of the data based on the professional position shows that physicians in a leading position rate the implementation of certain measures higher than their colleagues in a subordinate position. For one thing, this phenomenon is certainly due to a subjective distortion of perception based on one’s own experience. For example, while chief physicians and senior physicians are more likely to perform minimally invasive procedures, residents and attending physicianss are more likely to perform open training procedures. Furthermore, it can be assumed that the information provided by respondents at higher levels is more likely to correspond to the desired procedure, while the information provided by Residents and Attending physicians corresponds to the procedure actually practised in everyday clinical practice. In addition to in-hospital resources such as mPOM assistants, SOPs and interdisciplinary mPOM teams, external support can be helpful to drive the implementation of mPOM concepts. Different companies offer support during the implementation process and, if needed, also feedback programs to monitor the continuation of the introduced measures after the implementation phase is completed. This can lead to a significant improvement in adherence, but industry-supported implementation processes are usually very cost-intensive and hospital/patient data must be shared with the companies.

A neutral and cost-free option on the other hand will be the freely accessible publication of the German S3 guideline on the perioperative management of gastrointestinal tumors (POMGAT guideline) [14]. Additionally, a new working group of the DGAV, the Surgical Working Group on Perioperative Care in Visceral Surgery (CA PeriVis), was founded to disseminate these evidence-based recommendations and increase implementation. To overcome the difficulties in implementing mPOM concepts, the most important goal of this working group is to establish a nationwide interdisciplinary and interprofessional training program as well as an audit and certification process.

### Limitations

The limitations of our survey include the hardly avoidable “non-response bias”. Since there are overlaps, particularly with regard to the membership of the DGAV and DGK, it is not possible to give an exact response rate, but we can assume a response rate of less than 5%. The reason for the low participation remains unclear, but unfortunately coincides with a similar survey among members of the German Society for Anaesthesiology and Intensive Care Medicine [28].

It is conceivable that only surgeons with an established perioperative concept answered the questionnaire, while other surgeons without an mPOM concept did not participate. This again promotes a social bias in our results. However, as all levels of training as well as different care settings were represented, we believe that the collective of participants can nevertheless be considered representative.

### Supplementary Information

Below is the link to the electronic supplementary material.

Supplementary file1 (DOCX 20 KB)

## Data Availability

The data that support the findings of this study are available on request from the corresponding author.
