# Supplementary material for: Adherence to fast track measures in colorectal surgery—a survey among German and Austrian surgeons
Source: Int J Colorectal Dis. 2023 Mar 25;38(1):80. doi: 10.1007/s00384-023-04379-9 (PMC10039823; doi:10.1007/s00384-023-04379-9)
Supplement: Supplementary file 1 — Supplementary file1 (DOCX 20 KB) [file 384_2023_4379_MOESM1_ESM.docx]

# **Perioperative medicine in colorectal surgery**

**Q1: Does your hospital have a standardised FAST track / ERAS treatment concept with required perioperative measures?**

- Yes, supported by the ERAS-Society
- Yes, supported by the industry
- Yes, without support
- No
- Other (please specify)

**Q2: If your hospital has an interdisciplinary Fast Track / ERAS team, which disciplines are represented in this team?**

- Surgery
- Anaesthesia
- Nurse specialised in Fast-Track / ERAS
- Nutritional medicine / nutritional counselling
- Physiotherapy
- There is no interdisciplinary team
- Other (please specify)

**Q3: Does your hospital have an SOP (Standard Operating Procedure) or comparable for the perioperative treatment of patients undergoing colorectal resections?**

- Yes
- No
- Other (please specify)

**Q4: Are your patients specifically informed about the perioperative treatment concept for colorectal surgery as part of the outpatient preparation?** (Multiple selection possible)

- Yes, as part of the surgical risk education:
- Yes, by specialised nursing staff
- Yes, in the form of a patient brochure / information video etc:
- No
- Other (please specify)

**Q5: For which of the following preoperative measures do you offer your patients support, e:g: in the form of advice, training or prescription?** (Multiple selection possible)

- Nicotine abstinence
- Alcohol abstinence
- Increasing physical activity
- Optimisation of food intake / prevention of malnutrition
- Psycho(onco)logical care
- Other (please specify)

**Q6: Do your patients receive preoperative bowel preparation before colorectal surgery?**

- Only colon resections
- Only rectal resections
- Any elective colorectal resections
- No
- Other (please specify)

**Q7: If yes, which method of bowel preparation is performed in your clinic for colorectal resections?**

- Mechanical bowel preparation (colonic irrigation)
- Oral antibiotic bowel preparation
- Combination of mechanical and oral antibiotic bowel preparation
- Other (please specify)

**Q8: What is the average preoperative fasting period of your patients before colorectal surgery?**

- Please enter the average number of hours between the last intake of solid food and the operation
- Please enter the average number of hours between drinking clear liquids and the operation

**Q9: Which surgical procedure is predominantly used for colon resections at your hospital?**

- Robotic resection
- Laparoscopic surgery
- Open surgery
- Other (please specify)

**Q10: Which surgical procedure is predominantly used at your clinic for rectal resections?**

- Robotic resection
- Laparoscopic surgery
- Open surgery
- Other (please specify)

**Q11: Which measures for post-operative pain therapy are mainly used in colorectal surgery at your clinic?**

*For open surgery*

- Epidural analgesia via PDK
- TAP block
- Local infiltration of the surgical access
- Analgesic pump / Patient controlled analgesia
- Short infusions / oral administration of opioids
- Analgesia using only non-opioid analgesics

*For minimally invasive procedures*

- Epidural analgesia via PDK
- TAP block
- Local infiltration of the surgical access
- Analgesic pump / Patient controlled analgesia
- Short infusions / oral administration of opioids
- Analgesia using only non-opioid analgesics

**Q12: When is the postoperative removal of the following drains/ accesses after colorectal surgery usually performed?**(Please select one of the following options for both colon and rectal resection from the drop-down menu)

*Urinary catheter*

- Postoperatively in the operating room
- On the first postoperative day
- On the 2nd-5th postoperative day
- After mobilisation is completed
- Other

*Gastric tube*

- Postoperatively in the operating room
- On the first postoperative day
- On the 2nd-5th postoperative day
- After mobilisation is completed
- Other

*Abdominal drains*

- No drains are inserted
- On the first postoperative day
- Dependent on the amount of discharge
- Other

*Central Lines (Central Venous Catheters)*

- It is not used regularly
- On the first postoperative day
- On the 2nd-5th postoperative day
- Other

**Q13: Please indicate on which postoperative day after colorectal surgery, given a normal postoperative course, the following measures are performed? (The day of surgery is day 0)**

- Mobilisation to the edge of the bed/ to a chair
- Mobilisation to the ward corridor
- Consumption of liquid food
- Consumption of solid food
- Termination of intravenous fluid substitutions

**Q14: Do your patients regularly receive a laxative postoperatively after colorectal surgery?**

- Yes, always on the first post-operative day
- Yes, always on the second post-operative day
- Yes, always on the third post-operative day
- Yes, if there is no bowel movement within the first five postoperative days
- No
- Other (please specify)

**Q15: Are your patients monitored postoperatively in an intensive care unit in case of regular intraoperative course?**

- Only colon resections
- Only rectal resections
- Only patients who have had open surgery
- Yes, regardless of the operation performed
- Not regularly
- Other (please specify)

**Q16: What is the average post-operative hospital stay at your hospital?**
(Please select one of the following options for both colon and rectal resection from the drop-down menu)

- 1 - 3 days
- 4 - 6 days
- 7 - 9 days
- 10 -12 days
- 13 - 15 days
- 16 - 18 days
- 19 days or longer

**Q17: How would you describe the cooperation regarding perioperative management with the anaesthesiological partner on a scale from 1 (very good) to 6 (insufficient)?**

**Q18: How old are you?**

- 30 years or younger
- 31-40 years
- 41-50 years
- 51-60 years
- 61-70 years
- Older than 70 years

**Q19: What is your professional position?**

- Residency
- Resident
- Senior physician
- Leading Senior Physician
- Chief of Surgery
- General practitioner
- Other (please specify)

**Q20: Where are you currently working?**

- University Hospital
- Tertiary care hospital
- Secondary care hospital
- Primary care hospital
- Individual practice / group practice / joint practice
- MVZ
- Other (please specify)

**Q21: Your comments on this survey**
